# Viral Dark Matter: Illuminating Protein Function, Ecology, and Biotechnological Promises

**DOI:** 10.1021/acs.biochem.5c00349

**Published:** 2025-11-20

**Authors:** James C. Kosmopoulos, Karthik Anantharaman

**Affiliations:** † Department of Bacteriology, 312674 University of Wisconsin-Madison, Madison, Wisconsin 53706, United States; ‡ Microbiology Doctoral Training Program, University of Wisconsin-Madison, Madison, Wisconsin 53706, United States; § Department of Integrative Biology, University of Wisconsin-Madison, Madison, Wisconsin 53706, United States; ∥ Department of Data Science and AI, Wadhwani School of Data Science and AI, Indian Institute of Technology Madras, Chennai, Tamil Nadu 600036, India

## Abstract

Viruses are the most abundant biological entities on Earth and play central roles in shaping microbiomes and influencing ecosystem functions. Yet, most viral genes remain uncharacterized, comprising what is commonly referred to as “viral dark matter.” Metagenomic studies across diverse environments consistently show that 40–90% of viral genes lack known homologues or annotated functions. This persistent knowledge gap limits our ability to interpret viral sequence data, understand virus-host interactions, and assess the ecological or applied significance of viral genes. Among the most intriguing components of viral dark matter are auxiliary viral genes (AVGs), including auxiliary metabolic genes (AMGs), regulatory genes (AReGs), and host-physiology-modifying genes (APGs), which may alter host function during infection and contribute to microbial metabolism, stress tolerance, or resistance. In this Review, we explore recent advances in the discovery and functional characterization of viral dark matter. We highlight representative examples of novel viral proteins across diverse ecosystems, including human microbiomes, soil, oceans, and extreme environments, and discuss what is known and still unknown about their roles. We then examine the bioinformatic and experimental challenges that hinder functional characterization and present emerging strategies to overcome these barriers. Finally, we highlight both the fundamental and applied benefits that multidisciplinary efforts to characterize viral proteins can bring. By integrating computational predictions with experimental validation and fostering collaboration across disciplines, we emphasize that illuminating viral dark matter is both feasible and essential for advancing microbial ecology and unlocking new tools for biotechnology.

## Graphical abstract

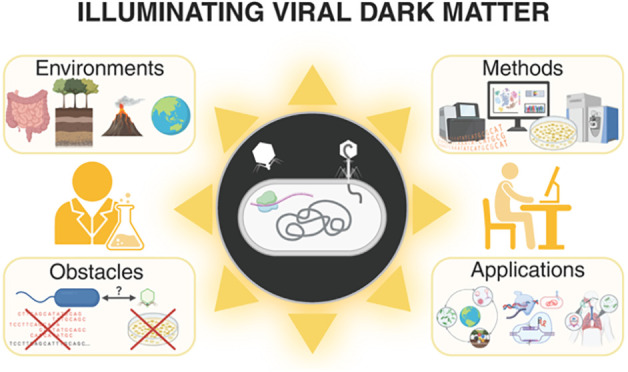

## 1 Introduction

Viruses are the most numerous biological entities on Earth, infecting organisms across all domains of life and shaping the structure and function of virtually every ecosystem.^1–3^ Viruses are incredibly diverse, containing single-stranded or double-stranded DNA and RNA genomes, infecting all domains of life, and employing vastly different reproductive strategies that all depend on a host organism to replicate their genomes for them.^4,5^ Among this diversity, bacteriophages (phages; viruses that infect bacteria) are the most abundant in nature. Phages manipulate their bacterial hosts over the course of infection with profound consequences on microbiomes.^6^ This manipulation is often achieved by genes that augment host cellular processes but are auxiliary to essential virus functions, such as genome replication, capsid assembly, and/or lysis. These auxiliary viral genes (AVGs)^7^ can include genes that augment (1) host metabolism (auxiliary metabolic genes, or AMGs) such as genes encoding photosynthesis subunits like *psbA*, *psbD*, and *hli*,^6,8–10^ (2) host physiology (auxiliary physiology genes, or APGs) with genes such as ones encoding sporulation proteins like *spo0A*, *spoIID*, *spoIIID*, and *spoVI*,^11^ and (3) host gene regulation (auxiliary regulatory genes, or AReGs), with the key difference from AMGs and APGs being that they can interfere with the regulation of host gene expression beyond directly interacting with metabolic or physiological pathways, such as (anti-) sigma factors like *asiA* ^12^ or transcriptional regulators like *luxR*.^13^ The unifying feature of the three types of AVGs is that when expressed, AVGs “reprogram” key host functions to ultimately benefit phage reproduction.^14–16^

At the cellular scale, AMGs may boost energy production^16–18^ and AReGs may upregulate the expression of host-encoded translation machinery,^19^ both to support virus reproduction. Additionally, APGs may inhibit host sporulation and dormancy to maintain favorable conditions for infection.^20^ When such interactions scale across microbial communities, they can drive global shifts in ecosystem function and microbial evolution.^21–25^ Yet, beyond a limited number of well-characterized examples, we do not know the diversity of ways in which phages manipulate their hosts due to our lack of ability to annotate viral proteins.

Owing to viral genomic diversity and rapid evolution, our ability to assign functions to viral proteins by sequence similarity is severely limited. Environmental surveys consistently show that a large fraction of viral genes lack functional annotation. In environmental studies, 40–90% of viral DNA sequences (mostly encoded by dsDNA phages and sometimes dsDNA eukaryotic viruses, since common sequencing techniques often bias toward dsDNA viruses, see Section 2) cannot be assigned to known functions or even align to previously described viral sequences,^26–29^ a phenomenon often termed “viral dark matter.” Notably, recent advances in sequencing ssDNA and RNA viruses reveal that these groups also harbor extensive dark matter,^30–32^ potentially exceeding that of dsDNA viruses, highlighting that the challenge extends across all viral genome types. Even in curated databases of reference viral genomes (including phages and viruses of archaea and eukaryotes), roughly 40–45% of proteins^33,34^ and 75–85% of protein families are annotated as hypothetical or unknown (Figure 1). The gap between viral gene discovery and functional characterization is widening: modern metagenomic studies are uncovering millions of new viral genes and genomes, yet most of these encode proteins of unknown function.^35^ For example, the largest annotated public database of viruses, IMG/VR v4, now contains >15 million viral sequences,^35^ and a recent human gut viral catalog added >450,000 new viral protein clusters (92% previously undocumented).^32^ This accumulation of viral proteins with undefined roles represents a major bottleneck in understanding virus-host interactions. Crucially, most viral gene functions inferred from sequence remain putative until experimental validation, leaving many predicted roles unconfirmed in the lab.

**Figure 1:**
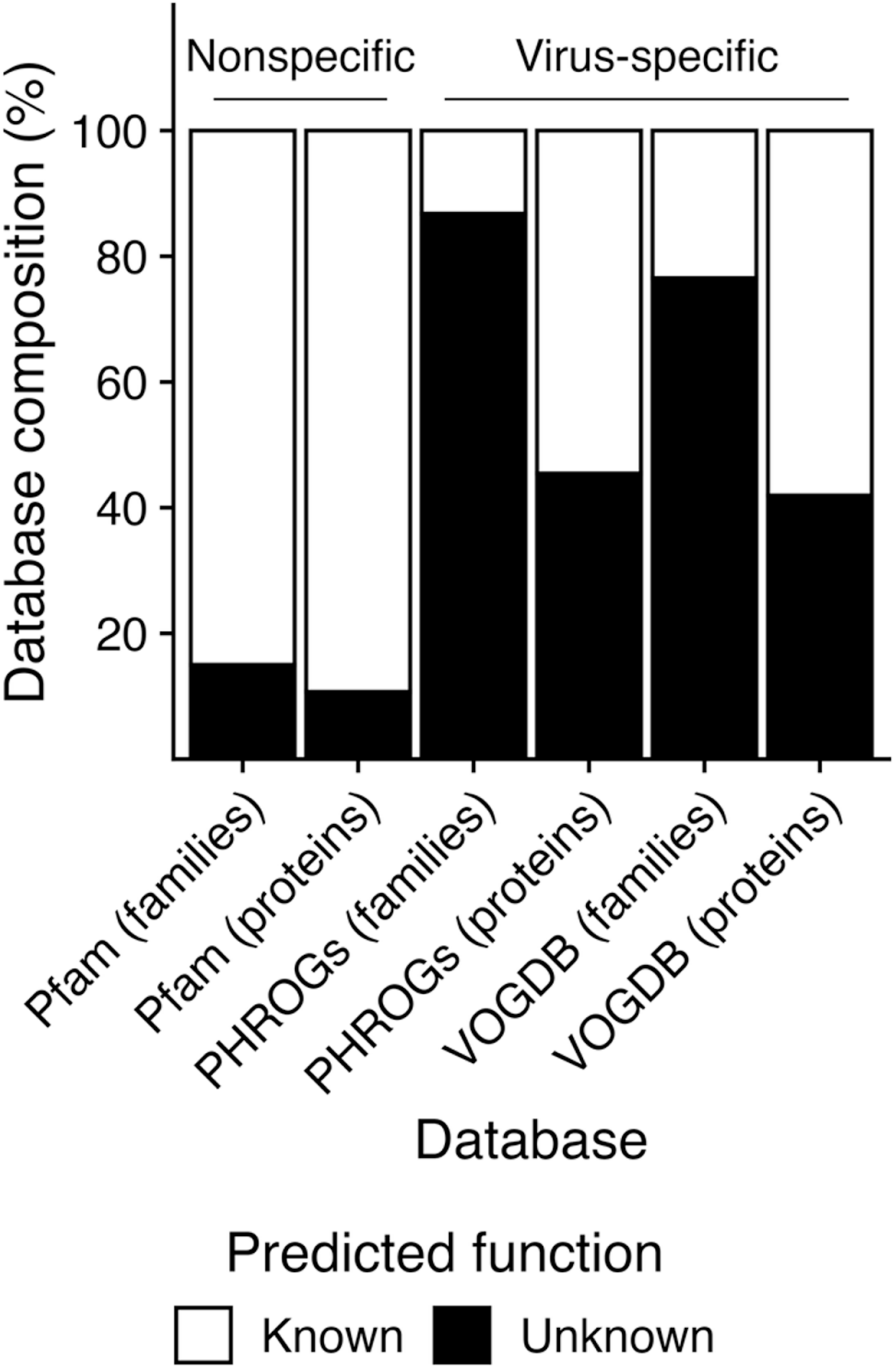
Prevalence of proteins with unknown functions in public databases. Protein families are clusters of multiple homologous proteins or sometimes an individual protein “singleton” with no other known homologues. Bars display the database composition at the family level (% of families) and protein level (% of all proteins in the database, irrespective of their family assignments). Families or proteins without functional annotations, or annotated with “hypothetical protein,” “domain of unknown function”, or “protein of unknown function,” were considered to have “unknown” functions; otherwise, they were considered to have “known” functions. The Prokaryotic Virus Remote Homologous Groups (PHROGs)^34^ database and Virus Orthologous Groups Database (VOGDB)^33^ contain only proteins and protein families of viral origin and thus are labeled as “virus-specific.” The Pfam^36^ database contains proteins from diverse biological entities and organisms (viruses as well as eukaryotes, bacteria, archaea, and other mobile genetic elements, etc.) and is thus labeled “nonspecific.”.

Why is viral “dark matter” so pervasive? A key reason is that the majority of microbes (and thus their viruses, which need to infect microbes to reproduce) cannot be readily cultured in the lab. For cellular microbes, metagenomic sequencing has revealed that cultivated isolates represent under 10% of total microbial biodiversity, with the rest coming from uncultivated lineages.^37^ Viruses face a similar gap–the vast majority of known viral diversity has been uncovered by cultivation-independent methods only.^35^ Additionally, viral genomes and proteins often evolve rapidly and share little sequence similarity to known references, hindering homology-based annotations.^29^ Many viral genes are small or novel open reading frames (ORFs) with weakly conserved domains,^38,39^ and some functions may be context-dependent (e.g., acting only under specific host or environmental conditions),^40,41^ making them hard to predict computationally. As a result, standard genome annotation pipelines assign generic labels like “hypothetical protein” to roughly half or more of viral genes in a novel genome sequence.^33,34^ Overcoming this functional unknown is critical: without understanding what these viral proteins do, we miss key insights into viral ecology, their roles in microbial metabolism, and potential applications in phage therapy and biotechnology.

Characterizing the functions of these unknown viral proteins holds considerable promise for both ecological understanding and practical applications. From an ecological standpoint, viruses are pivotal regulators of microbial populations and nutrient transformations.^2,6^ Uncharacterized viral genes could be mediating metabolic processes like carbon, nitrogen, and phosphorus transformations, or host–microbe interactions in ways we have yet to recognize. Every new function illuminated within viral dark matter can reveal novel mechanisms by which viruses impact host metabolism or shape ecosystem interactions and dynamics. From an applied perspective, viral genomes represent a vast reservoir of novel enzymes and bioactive molecules. Indeed, phage-derived proteins have already provided invaluable tools in biotechnology (for instance, various DNA polymerases and ligases used in molecular biology originate from phage genes)^42^ and potent antibacterial agents (e.g., phage lytic enzymes in phage therapy).^43,44^ It stands to reason that many of the currently unknown viral proteins could similarly be harnessed for biotechnological innovation or as therapeutics. In short, shining light on viral dark matter will deepen our understanding of how viruses are microbial ecosystem engineers and may also uncover new enzymes for bioengineering and medicine.

In this review, we examine the emerging efforts to illuminate viral dark matter, the vast repertoire of viral proteins with unknown functions. First, we describe how modern “omics” techniques have been used to discover viral dark matter, emphasizing how these approaches detect viruses and their proteins without the need for culturing. Next, we focus on what these methods have revealed, highlighting the functional insights gained into viral proteins across diverse ecosystems, from soils to the human gut, oceans, and extreme habitats, and discussing how these discoveries have expanded the known virosphere. We then turn to the major obstacles to functional characterization of viral genes, including bioinformatic limitations and experimental challenges, to clarify why progress remains difficult. Finally, we explore emerging strategies and technologies aimed at overcoming these hurdles, emphasizing cross-disciplinary approaches that connect computational predictions to laboratory validation. By synthesizing what is “known about the unknowns” for viruses and outlining a roadmap for their characterization, our aim is to accelerate the integration of viral dark matter into more biochemical studies to broaden our collective understanding of microbiomes.

## 2 ‘Omics-Based Discovery of Viral Dark Matter

Early efforts to mine microbial genomes had identified thousands of viral genomes integrated in bacterial/archaeal genomes (prophages), providing the first viral representatives for dozens of new microbial phyla.^26^ In one early study, Roux et al. (2015) recovered ∼12,500 viral genomes from publicly available microbial genomes, including viruses infecting 13 bacterial phyla that were previously unsampled at the time.^26^ Later efforts focused on discovering viral genomes not just from cultivated bacterial/archaeal genomes but from entire microbial communities (metagenomics). By directly sequencing DNA from environmental or host-associated samples, metagenomics can uncover an enormous diversity of viruses (Table 1), including many that infect uncultivated microbes.

**Table 1:** ‘Omics-Based Methods Used to Study Viral Proteins, Their Benefits, and Drawbacks

method	starting molecule	benefits	drawbacks	examples
Metagenomics	DNA (environmental or host-associated DNA)	- Captures viral genomes directly from samples without the need for culturing hosts or viruses, enabling the discovery of vast numbers of new viruses (including those infecting uncultivated microbes)	- Biased to DNA viruses: Fails to detect RNA viruses, necessitating metatranscriptomics for RNA virus discovery	- 11.8 million protein-coding genes identified in 190,000 DNA virus genomes from human gut metagenomes, 75% of which had unknown functions and 45% had no similarity to proteins in other databases^32^
		- Reveals the genetic capacity of viruses: uncovers viral genes that may hint at virus-host interactions	- Provides limited insight into activity: DNA presence does not indicate if a viral gene is expressed or functional in the environment	- 87,000 virus-encoded auxiliary metabolic genes encoded by 690,000 dsDNA virus genomes from global ocean metagenomes^21^
Metatranscriptomics	RNA (total community RNA, often after rRNA depletion)	- Detects actively expressed viral genes as RNA transcripts, highlighting which viral functions are in use *in situ*	- RNA is less stable: Viral RNA can be rare and easily degraded; samples often require careful processing and enrichment	- 52 auxiliary viral genes, encoded by 2700 RNA viral genomes predicted to infect fungal, ciliate, and invertebrate hosts from soil RNA metatranscriptomes^30^ (few studies^31^ were previously able to identify auxiliary genes encoded by RNA viruses in environmental samples)
		- Enables the discovery of RNA viruses that lack DNA stages and are invisible to DNA metagenomics	- Biased toward current infections: Only detects viruses that are actively transcribing. Dormant viruses or DNA viruses with no ongoing transcription in the sample will be missed, potentially underestimating total viral contributions	- Structural, nucleic acid-binding, galactose-binding, nuclease, hydrolase, and uncharacterized domains were identified in 647,000 proteins encoded by 370,000 RNA viral genomes recovered from a large collection of environmental metatranscriptomes^45^
Metaproteomics	Proteins (all proteins extracted from a community sample)	- Confirms protein-level expression of viral genes: Verifies that hypothetical ORFs from viral genomes are translated and allows functional inferences	- Low sensitivity for viruses: viral proteins are often low-abundance amid a vast host protein background, so metaproteomics may preferentially detect only the most abundant viral proteins	- Nearly 1900 viral proteins detected in oceans from metaproteomics revealed highly conserved and expressed capsid proteins, but over one-third were still not able to be assigned potential functions^27^
		- Complements genomic data by assigning tentative functions to unknown viral proteins based on detected peptides	- Technical complexity: environmental proteomics is experimentally and computationally demanding, needing extensive sample processing, high-end mass spectrometry, and complex peptide-to-protein matching pipelines	- Metaproteomics of ruminant microbiomes revealed 64 viral proteins expressed by 53 viral populations, 80% of which had no identifiable functions, with the remaining being structural proteins^46^

Public databases of viral genomes have expanded dramatically with the advent of metagenomics. The IMG/VR v4 database, released in 2022, now contains over 15 million viral genomes or genome fragments, the vast majority derived from metagenomes.^35^ This represents a 6-fold increase compared to the previous release from 2021,^47^ highlighting the explosion of new viral sequences discovered from metagenomics in a relatively short period of time (Figure 2). In IMG/VR v4, viral genomes originating from metagenomes outnumber cultivated isolate genomes by 2 orders of magnitude (Figure 2), demonstrating that metagenomics captures far more viral diversity than cultivation-dependent methods. Metagenomic surveys across diverse environments, including the human gut, soils, oceans, and other habitats, continue to expand the known virosphere. For instance, recent human gut virome studies alone have each discovered tens or hundreds of thousands of viral genomes, many with no close matches in existing virus databases.^32,48–50^ Metagenomics has thus revolutionized the discovery of viruses and their genes, bypassing the need for the culturing of hosts or viruses.

**Figure 2:**
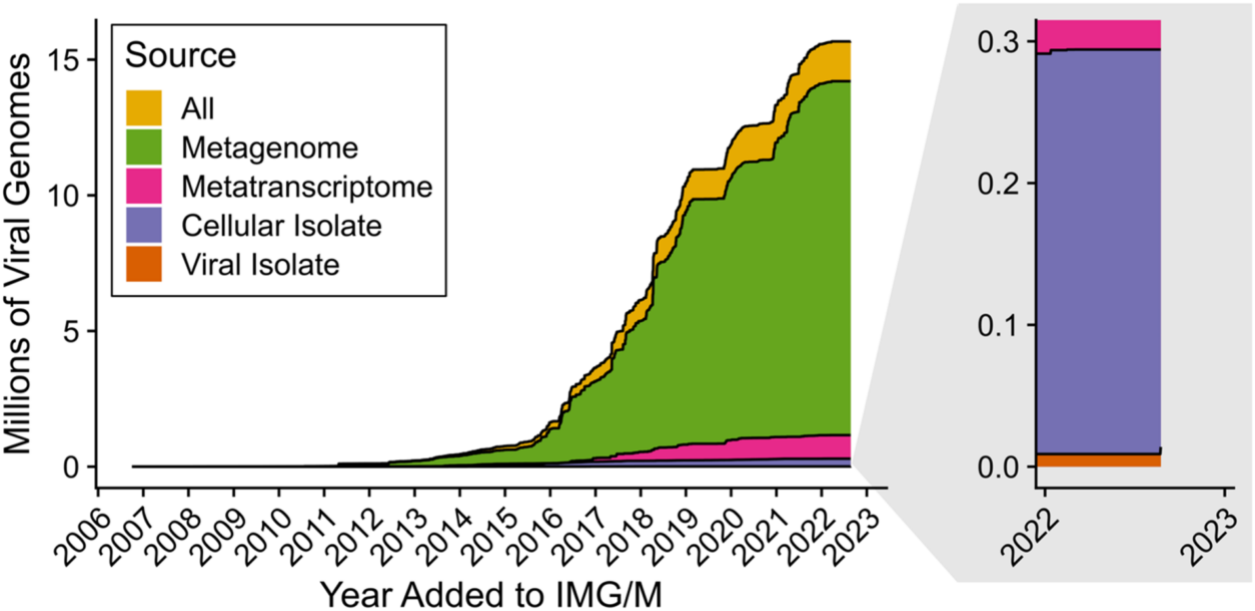
Number of viral genomes added to the Integrated Microbial Genomes and Microbiomes (IMG/M) Database over time. The cumulative sum of the number of viral genomes (in millions of sequences) originating from metagenomes, metatranscriptomes, cultivated isolate or single-cell bacterial, archaeal, and eukaryotic genomes (cellular isolate), and cultivated isolate or single-cell viral genomes (Viral Isolate), and all sources together, are provided. To determine the dates that viral genomes were added to IMG/M and the source of their sequences, metadata for each genome in IMG/VR v4 was mapped to their corresponding IMG/M and JGI Genomes Online Database (GOLD)^51^ “NCBI Domain”, “Sequence origin (doi)”, “GOLD Analysis Project Type”, “Genome Name/Sample Name”, and “Add Date” fields using matching “Taxon_oid” entries.

While gene catalogs from metagenomics outline the scope of viral dark matter, complementary “omics” approaches can add an additional dimension by identifying which viral genes are active in nature and by identifying uncultivated RNA viruses. Metatranscriptomics (the bulk sequencing of RNA from entire microbiomes) has emerged as a powerful tool for discovering RNA virus genomes (Table 1), providing insights into their encoded genes and potential ecological roles that remain elusive from DNA-based studies alone. Although the number of viral genomes uncovered by metatranscriptomics is only a fraction of those identified using metagenomics (Figure 2), recent metatranscriptomic analyses have significantly expanded our understanding of RNA virus diversity, particularly in understudied soil environments. For instance, Starr et al. employed metatranscriptomics to reconstruct the RNA viral community across multiple soil habitats, uncovering thousands of novel RNA viruses primarily from the *Narnaviridae* and *Leviviridae* families.^52^ Similarly, Hillary et al. identified thousands of RNA viral sequences across different grassland soils using RNA viromics (metatranscriptomics enriched for virus-like particles), demonstrating the prevalence of diverse RNA viruses infecting not only bacteria but also fungi, plants, vertebrates, and invertebrates.^53^ Notably, a significant fraction of these viruses belongs to previously understudied groups. Similarly, Wu et al. and Pratama et al. utilized metatranscriptomics to explore RNA viruses in permafrost soils undergoing thaw due to climate change, with each study revealing thousands of novel RNA viruses encoding AVGs potentially involved in nutrient transformations and host metabolism.^30,31^ Together, these studies emphasize RNA viruses as key yet largely uncharacterized contributors to viral dark matter, underscoring the need for continued functional investigations into RNA-virus encoded proteins.

Going a step further from DNA- and RNA-based inferences, direct analyses of translated viral proteins have been particularly illuminating. Metaproteomics (mass-spectrometry-based protein identification in environmental samples) has been used to detect microbially encoded proteins in complex communities (Table 1). These efforts have confirmed the production of many hypothetical proteins and generated new hypotheses about the functions that viruses contribute to or disrupt in microbiomes.^54,55^ A landmark study by Brum et al. (2016) applied metaproteomics alongside DNA sequencing to ocean water samples, identifying 1875 virus-associated structural proteins from uncultivated marine viruses.^27^ Remarkably, over half of these proteins had been previously unannotated but were assigned broad functional categories through their approach, such as “capsid protein” or “tail protein”. The most abundant proteins in their data sets turned out to be components of viral capsids, suggesting that a particular conserved capsid fold may be among the most abundant biological structures known to science.^27^ More metaproteomic studies that included viruses in human,^54^ ruminant,^46^ and soil^56^ microbiomes discovered more previously unknown virus-encoded proteins with metabolic functions that have the potential to impact their entire communities. However, virus-focused applications of metaproteomics remain far fewer than studies employing the other approaches discussed above, likely due to substantial methodological challenges for viral metaproteomics (Table 1). Despite these limitations, existing studies collectively demonstrate that proteomics can illuminate portions of viral dark matter: by directly observing proteins, researchers proposed functions for hundreds of widespread and conserved viral genes that were previously unknown sequences and were thus able to propose new frameworks for microbial and viral functions in microbiomes.

Together, culture-independent metagenomics, metatranscriptomics, and metaproteomics have dramatically improved our understanding of the functional diversity of viruses. They enable high-throughput discovery of novel genomes, genes, and proteins that can guide further study. Still, although assigning potential functions for proteins encoded by uncultivated viruses has been a big leap, specific biochemical activities of these proteins remained unverified in many cases, highlighting the need for deeper functional analysis to move from functional predictions to proof.

## 3 Viral Dark Matter Across Diverse Ecosystems

### 3.1 Human Gut Microbiome

Viruses are integral to human microbiomes, with the gut virome being particularly rich in phages. Large-scale metagenomic projects have revealed tens of thousands of distinct phage species in the human gut, most of which were previously unknown.^32,48–50^ A 2021 study of human gut DNA viruses by Nayfach et al. (mostly dsDNA phages and other DNA viruses with unknown host taxonomy) clustered ∼11.8 million viral genes into 459,375 protein clusters, finding that 45% of the genes had no matches to any known profiles and another 30% matched only profiles of unknown function.^32^ In other words, ∼75% of human gut viral genes currently lack a clear functional annotation. Interestingly, only 39% of the 459,375 protein clusters were singleton proteins, suggesting that the remaining 61% have homologues in the human gut. This means that although most viral genes in the human gut have no currently known function and appear as viral dark matter, they are still abundant and conserved, suggesting that they are functionally important to the human gut microbiome overall and make them promising candidates that warrant further study. Of the proteins that were able to be assigned putative functions, some of the largest protein clusters included phage structural proteins, DNA packaging, replication, and binding proteins, lysis proteins, and, interestingly, reverse transcriptases, which suggested that diversity-generating retroelements (DGRs) are highly prevalent in the human gut and contribute to virus-host coevolutionary dynamics. Some β-lactamases were also found to be encoded by human gut viruses, suggesting that a portion of viral dark matter in the human gut may be involved in antimicrobial resistance, although this is thought to be a rare phenomenon.^57^

Despite the large fraction of viral proteins in the human gut with unknown functions, some metabolic or ecological roles of gut phage genes are beginning to emerge from metagenomic data (Table 2), hinting at the potential functions of viral dark matter. Bacteriophages in the gut have been found to carry auxiliary genes that could influence the bacterial host’s fitness or metabolism. For instance, gut phages sometimes encode auxiliary carbohydrate-active enzymes that might help their hosts degrade mucin in the gut lining or dietary polysaccharides.^58,59^ In another study by Kieft et al. (2021), viruses encoding enzymes involved in assimilatory and organic sulfur metabolism were ubiquitous in many environments, especially the human gut.^60^ They also experimentally demonstrated that increasing concentrations of sulfide (H_2_S), a byproduct of the viral cysteine degradation enzymes, was met with an increase in viral population sizes, which can have far-reaching implications on the overall gut microbiome and human health.^60^ Beyond metabolism, human gut phages may influence host life cycles. Recent studies noted that human gut phages harbor genes involved in bacterial sporulation,^11,20^ and are even functional in inhibiting host sporulation.^20^ However, while some cases highlighted here experimentally validated the activity of phage genes (Table 2), the activity of most phage-encoded proteins identified across these studies remains to be demonstrated experimentally. The human gut virome thus contains a myriad of novel genes that potentially affect host physiology, gene regulation, and metabolism, but confirming these functions still requires targeted experiments.

**Table 2:** Examples of Auxiliary Genes Encoded by Viruses and Their Functions^a^

environment	genes/proteins	function	validation	citation
Human gut	Transcriptional repressor (*lexA*)	Bacterial sporulation	Not validated	Schwartz et al.^11^
	Stage 0, II, III, V sporulation proteins (*spo0A spoIID spoIIID spoIIIE spoVG*, *spoVT*)			
	*sigF*- and *sigG*-like sigma factors	Bacterial sporulation	Heterologous expression in *Bacillus subtillis* and RNA-seq	Schwartz et al.^20^
	Reverse transcriptase	Diversity-generating retroelements	Not validated	Nayfach et al.^32^
	Alginate lyase	Mucin degradation	Plaque assay on native *Pseudomonas aeruginosa* host, viscosity reduction, and reducing-sugar assay	Glonti et al.^59^
	Cysteine synthase (*cysK*)	Sulfide production from cysteine degradation	*Lactococcus lactis* phage-host model, RNA-seq, untargeted mass spectrometry	Kieft et al.^60^
Soil	O-acetylhomoserine [thiol]-lyase (*cysD*)	Assimilatory sulfate reduction	Not validated	Kieft et al.^60^; Rodríguez-Ramos et al.^56^
	Phosphoadenosine phosphosulfate reductase (*cysH)*	Assimilatory sulfate reduction	Not validated	Kieft et al.^60^
	Sulfate adenylyltransferase subunit/adenylylsulfate kinase (*cysNC*)			
	Phosphohistidine phosphatase (*sixA*)	Central carbon metabolism	Not validated	Trubl et al.^63^
	Chitosanase (GH75)	Chitin degradation	Gene synthesis and recombinant expression in *Escherichia coli*	Wu et al.^65^
	Cytochrome C oxidase cbb3-type subunit III (*ccoP*)	Energy metabolism	Stable isotope probing (SIP) metagenomics	Trubl et al.^69^
	E1-E2 ATPase			
	GTP cyclohydrolase			
	Multicopper oxidase			
	NAD-dependent epimerase/dehydratase			
	α -mannosidase (GH5)	Hemicellulose degradation	Gene synthesis and recombinant expression in *E. coli*	Emerson et al.^64^
	UDP-galactose-4-epimerase (*galE*)	Organic nitrogen or carbon utilization	Not validated	Rodríguez-Ramos et al.^56^; Richy et al.^66^
	L-2-haloacid dehalogenase (L-DEX)	Organochlorine pesticide degradation	PCR-based accurate synthesis and recombinant expression in E. coli	Zheng et al.^68^
	Pectate lyase (PL1)	Pectin degradation	Not validated	Rodríguez-Ramos et al.;^56^; Richy et al.^66^
	Phosphate starvation-inducible protein (*phoH*)	Regulation of phosphorus uptake	Not validated	Han et al.^62^
Aquatic	Adenylylsulfate kinase (*cysC*)	Assimilatory sulfate reduction	Not validated	Kieft et al.^60^
	Ammonia monooxygenase subunit C (*amoC*)	Nitrogen & energy metabolism	Not validated	Ahlgren et al.^70^
	Photosystem II P680 reaction center D1 and D2 proteins (*psbA*, *psbD*)	Photosynthesis	Microarray & RT-qPCR with *Procholoroccus* phage-host model	Sullivan et al.^71^; Lindell et al.^8^
	High-light inducible protein (*hli*)			
	Phosphate transport system substrate-binding protein (*pstS*)	Phosphate scavenging	Phosphate-uptake assay in native *Synechococcus* host; infection kinetics, RNA-seq, and heterologous expression in E. coli	Rihtman et al.^72^
	Ribose-5-phosphate isomerase B (*rpiB*)	Central carbon metabolism	Not validated	Tian et al.^21^
	Ribulose-phosphate 3-epimerase (*rpe*)			
	Transketolase (*tkaA*, *tktB*)			
	Transaldolase (*talA*, *talB*)			
	Fructose-bisphosphate aldolase (*fbaB*)			
	Fructose-1,6-bisphosphatase (*glpX*)			
	Catalase-peroxidase (*katG*)	Hydrogen peroxide decomposition	Not validated	Zhou et al.^22^
	Methane monooxygenase subunit C (*pmoC*)	Methane metabolism	Not validated	Zhou et al.^22^
Extreme	Arsenate reductase (*arsC*)	Arsenic metabolism	Not validated	Langwig et al.^73^
	(Reverse) dissimilatory sulfite reductase subunits A and C (*rdsr*, *dsrA*, *dsrC*)	Dissimilatory sulfate reduction	Not validated	Anantharaman et al.^74^; Kieft et al.^75^
	Adenylylsulfate reductase (*aprB*)	Dissimilatory sulfate reduction	Not validated	Langwig et al.^73^
	Cytochrome bd ubiquinol oxidase subunit I and II (*cydAB*)	Energy metabolism	Not validated	Langwig et al.^73^
	Pyruvate formate-lyase activating enzyme (*pflA*)	Fermentation	Not validated	Langwig et al.^73^
	Transcription factor (*whiB*)	Host stress response	Not validated	Hwang et al.^76^
	Transcriptional regulator (*luxR*)			
	Nitric oxide reductase subunit B (*norB*)	Nitrogen & energy metabolism	Not validated	Langwig et al.^73^
	Phosphate starvation-inducible protein (*phoH*)	Regulation of phosphorus uptake	Not validated	Langwig et al.^73^
	Sulfane dehydrogenase subunits C and D (*soxC*, *soxD*), *soxYZ* (Sulfur-oxidizing protein *soxYZ*)	Sulfur oxidation	Not validated	Kieft et al.^75^
	SSV9 B310, SSV11 p29	Toxin-antitoxin system	Infection assay, RNA-seq, and overexpression in native host *Sulfolobus islandicus*	DeWerff et al.^77^

a Examples include functions highlighted in Section 3: *Viral Dark Matter Across Diverse Ecosystems*, but are not exhaustive and do not list all putative auxiliary gene functions identified in each study. Examples marked as “not validated” in the “Validation” column indicate that the functions were predicted but not experimentally validated in the cited studies.

### 3.2 Soil Environments

Soils harbor some of the most complex viral communities. Metagenomic surveys of soils (including agricultural soils, grasslands, forests, wetlands, and permafrost) have revealed a wide diversity of DNA and RNA viruses. The majority of viruses detected and characterized from soil to date are dsDNA bacteriophages, although archaeal and eukaryotic viruses are also reported to a lesser extent and can include ssDNA, dsDNA, and RNA viruses. Importantly, the current extraction, sequencing, and analytical methods commonly used to study soil viromes tend to bias against ssDNA viruses, RNA viruses, and overall viruses of eukaryotes such as fungi unless measures are taken to sequence different molecules.^61^ Although the apparent dominance of dsDNA phages in soils reflects their biological prevalence (since bacterial DNA viruses are widespread and abundant overall^1–3^ ), this does not mean that non-dsDNA phages are always as rare as they may appear in current soil studies, and the same applies to environmental studies beyond soil as well.

As with microbiomes in any environment, a central question in soil viral ecology is how phages influence key metabolic pathways and biogeochemistry. Metagenomic studies of many soil types, agricultural fields, grasslands, forests, wetlands, and permafrost have revealed that 60–80% of predicted phage proteins lack functional annotation,^23^ consistent with the pervasiveness of viral dark matter globally, as mentioned in Section 1. Among the minority that could be annotated, many soil viruses were found to encode putative AMGs that could affect carbon, nitrogen, phosphorus, or sulfur nutrient transformations.^60,62–67^ For example, soil viromes contain genes for carbohydrate-active enzymes such as cellulases, chitinases, and lignin-degrading enzymes, which could influence the breakdown of organic matter in soils where they are enriched, such as in wetland and peatland soils.^52,56,63–65^ Some soil phages have also been found to encode genes involved in endospore formation.^63^ While these genes are not strictly viral “dark matter” because their functions are recognizable, they exemplify how genes that were once part of the dark matter can be linked to host metabolism, physiology, or stress responses. At the same time, a large fraction of soil viral coding sequences remains as uncharacterized proteins that may similarly augment host functions but cannot yet be assigned to known roles due to methodological limitations or extreme sequence divergence (see Section 4). Uncovering the roles of this residual viral dark matter is essential for understanding how viruses may further augment ecosystem-wide processes in soils.

Most functional annotations of soil phage genes remain based on sequence homology and remain mostly unvalidated; therefore, direct biochemical evidence of their activity is largely lacking. Nevertheless, their presence suggests that soil phages might enhance their hosts’ abilities to utilize complex nutrients or survive harsh soil conditions, thereby contributing to processes such as decomposition and nutrient turnover, if these phage genes are indeed expressed and functional during infection. In a recent compelling case confirming the activity of AMGs, Wu et al. isolated several candidate phage-encoded chitosanase genes from soil metagenomes and experimentally demonstrated their activity.^65^ Chitosanases break down chitin derivatives common in fungal cell walls and insect exoskeletons. Multiple viral chitosanase AMGs were expressed, and one gene product showed clear endochitosanase activity, confirming it as a functional enzyme. The researchers then crystallized the enzyme and solved its structure at ultrahigh resolution, providing a rare atomic structure of a soil viral AMG product.^65^ Further evidence comes from soils contaminated with organochlorine pesticides (OCPs). Zheng et al. demonstrated that viral genomes in OCP-contaminated soils harbored a higher abundance and diversity of AVGs associated specifically with pesticide degradation.^68^ Among these was a phage-encoded L-2-haloacid dehalogenase, which was experimentally validated to degrade OCP precursors, thereby alleviating pesticide toxicity and improving host bacterial growth.^68^

These works provide a proof of concept that viral dark matter in soils can be experimentally illuminated: genes predicted from metagenomes can yield active proteins with metabolic and biogeochemical roles (Table 2). They also underscore how little we have explored, with each study noting that their characterized enzymes were among the very few soil AVGs to ever be biochemically characterized. In summary, soil studies hint at diverse viral contributions to soil biochemistry (Table 2), but apart from rare attempts to study them in detail, such as the viral chitosanase and dehalogenase enzymes, most of these functions remain putative until confirmed by biochemical assays.

### 3.3 Aquatic Environments

Freshwater and marine ecosystems host an enormous diversity of phytoplankton-infecting viruses, including bacteriophages as well as DNA viruses of protists. These systems have been particularly important for showing how unknown functions hidden within viral dark matter can later be revealed as critical to ecosystem processes. Some of the first metagenomic studies of phage communities were conducted in marine environments,^78,79^ at a time when specialized bioinformatic tools were lacking and before biochemical experiments were conducted to test the functions of marine phage genes identified in metagenomes.^80^ From the outset, therefore, the challenge of viral dark matter was evident in marine viral ecology. Over time, as new computational approaches and targeted experiments emerged, some of the functional roles encoded by marine phages began to be illuminated, and the early discoveries were striking. Much of the previously uncharacterized sequence space proved to encode core viral functions such as DNA replication, virion formation, DNA packaging, and lysis.^71^ However, a notable fraction of the dark matter corresponded to unexpected functions, including genes involved in host-like processes such as carbon metabolism, often phylogenetically related to bacterial genes.^81^ These findings marked the discovery of auxiliary metabolic genes (AMGs),^9,82^ suggesting that many sequences now classified as viral dark matter in other environments may eventually be revealed as encoding for auxiliary functions with significant host- and ecosystem-level impacts.

The early efforts to study the functional capacity of aquatic viruses were especially influential, as much of what we know about AMGs today derives from ocean systems. Among the first examples were cyanophages that carry photosynthesis genes (*psbA*, *psbD*, *hli*, etc.),^6,8–10,71,83,84^ presumably to boost their host’s photosynthetic output during infection and thus increase energy availability for phage production. These photosynthetic AMGs are actively expressed, for instance, phage *psbA* transcripts increase during *Prochlorococcus* infection^8^ and have been shown to enhance host recovery from photoinhibition. Similarly, marine phages infecting autotrophs carry genes for nutrient acquisition. Cyanophages, for example, encode phosphate transporter genes like *pstS* to help their hosts scavenge phosphate,^72^ and viruses infecting ammonia-oxidizing archaea have been found to encode ammonia monooxygenase subunits (amoC), with viral copies being highly abundant and actively expressed in metagenomic samples,^70^ linking viral infection to the nitrogen cycle.

Marine phages also encode enzymes in core metabolic pathways such as glycolysis or the TCA cycle,^14^ highlighting phages’ broader metabolic reprogramming potential in central carbon metabolism. Recently, Tian et al. systematically cataloged AMGs encoded by dsDNA phages from Tara Oceans,^85^ a planetary-scale metagenomic sequence database sampled from diverse ocean ecosystems, identifying over 86,000 AMGs grouped into nearly 23,000 gene clusters, with 32% of the clusters having no matches in existing databases at the time.^21^ They mapped these AMGs to 128 metabolic pathways, highlighting lipid, nucleotide, and carbohydrate metabolism pathways as “hot spots” where viral gene copies either outnumbered their cellular counterparts or otherwise contributed to the majority of steps required for metabolic processes.^21^ Additionally, Zayed et al. (2021) conducted a detailed analysis of marine viruses (DNA phages and DNA viruses of eukaryotes) through an expanded viral HMM profile database (*efam*) and revealed tens of thousands of novel viral protein families, most lacking known functional annotations.^86^ This database, enriched by marine viral metaproteomic data, doubled the functional annotation rate of viral proteins compared with conventional methods, providing a powerful resource for future marine viral dark matter studies.

In freshwater systems, long-term studies further expand the scope of known AMGs. For example, Zhou et al. characterized over 1.3 million genomes of dsDNA phages and nucleocytoplasmic large DNA viruses of eukaryotes across a 20-year time series in Lake Mendota (Wisconsin, USA). In this study, Zhou et al. identified 574 AMG families, including genes involved in photosynthesis (*psbA*), methane oxidation (*pmoC*), and hydrogen peroxide decomposition (*katG*).^22^ Many of these AMGs were consistently active, suggesting stable roles in freshwater microbial metabolism over decades, despite substantial changes in environmental conditions and community composition.^22^ However, like marine ecosystems, most viral genomes and their encoded proteins in this study remained uncharacterized. Likewise, despite substantial progress in studying aquatic viruses, many viral genes are still uncharacterized due to the explosion of viral genes and genomes sequenced from the global oceans. For instance, Gregory et al. expanded marine viromes to include nearly 200,000 viral populations globally, yet most remained functionally unknown.^87^ Collectively, aquatic studies highlight that while some AMGs have been experimentally characterized and linked clearly to ecological processes (Table 2), the vast majority of viral-encoded proteins remain uncharacterized, emphasizing both the magnitude of viral dark matter and its potential to yield new AVGs and other ecologically important functions.

### 3.4 Extreme Environments

Viruses thrive even in extreme environments such as hot acid springs, hypersaline lakes, and deep-sea hydrothermal vents, often infecting extremophilic Bacteria and Archaea, and carrying unusual genes adapted to harsh conditions.^88^ These extreme microbiomes are rich in viral dark matter, partly because the host organisms themselves are genetically distant from well-studied model species.^88–90^ Viruses from these habitats encode proteins with distinct biochemical adaptations, such as thermally stable DNA polymerases and unique structural proteins capable of functioning under extreme temperatures, acidity, and salt concentrations.^90,91^ These extreme environments, therefore, may be a particularly unique reservoir of viral dark matter that encodes presently unknown functions, some of which may ultimately prove significant for both ecology and biotechnology due to their capacity to operate under extreme conditions.

Thermal environments, particularly hot springs, such as those in Yellowstone National Park (USA), provide striking examples of viruses uniquely adapted to extreme conditions. Metagenomic and single-cell genomic analyses have revealed extensive and complex networks of virus-host interactions in Yellowstone’s hot springs, where over 60% of microbial cells harbored viruses, often hosting multiple distinct viral types simultaneously.^92^ Many of these viruses infect thermophilic archaea like *S. islandicus* and exhibit remarkable genomic diversity.^90^ Intriguingly, these archaeal DNA viruses frequently encode toxin-antitoxin systems, whereby viral infection confers competitive advantages to their hosts by killing competing microbes.^77^ For instance, chronic infections by *Sulfolobus* spindle-shaped viruses (SSVs) mediate host fitness through virus-encoded toxins that were initially annotated as hypothetical proteins but were later shown by targeted experiments to act as secreted toxins.^77,93^ These toxins were shown to target uninfected, CRISPR-immune populations of competing archaea, and illustrated a form of virus-host mutualism that enables coexistence and shapes microbial community structure.^77,93^

Hypersaline and hyperarid environments, such as the Atacama Desert (Chile) and Great Salt Lake (Utah, USA), also harbor viruses that significantly influence microbiomes through encoded metabolic and stress-response genes. In microbial consortia inhabiting halite nodules of the hyperarid Atacama Desert, viruses infect diverse archaea and bacteria. Crits-Christoph et al. identified these viruses as crucial mediators of ecological interactions and microbial adaptation to high osmotic pressure.^91^ Complementing these findings, Hwang et al. demonstrated that Atacama viruses encode genes involved in microbial stress responses and spore formation, enhancing host resilience against extreme desiccation and radiation.^76^ Similarly, in hypersaline Great Salt Lake sediments, viruses carry AMGs associated with core metabolic processes, including photosynthesis, carbon fixation, formaldehyde assimilation, and nitric oxide reduction, directly linking viral genes to essential elemental and nutrient transformations.^94^

Viral contributions extend even to deep-sea hydrothermal vents, another extreme habitat characterized by high temperatures, high pressures, and chemical gradients. In these sulfur-rich habitats, sulfur-transforming AMGs are especially diverse and widespread. For example, Anantharaman et al. identified phages infecting marine chemolithoautotrophic bacteria that encode reverse dissimilatory sulfite reductase (*rdsr*) genes, enabling the conversion of elemental sulfur into sulfite at a key bottleneck in energy metabolism through sulfur oxidation.^74^ Similarly, Anderson et al. reported genomes of phages and archaeal viruses from vent ecosystems that carry AMGs involved in sulfur and methane metabolism.^95^ Kieft et al. further showed that AMGs such as *dsrA*, *dsrC*, *soxYZ*, and *soxCD* were not only widespread across hydrothermal ecosystems, but also highly expressed at levels several orders of magnitude higher than in background deep-sea samples.^75^ More recently, Langwig et al. identified thousands of uncharacterized phages and archaeal viruses from globally distributed hydrothermal vents, many encoding AMGs involved in arsenic, carbon, phosphorus, sulfur, and nitrogen transformations.^73^ These findings demonstrate that viruses directly contribute to microbial energy metabolism in hydrothermal vent ecosystems, highlighting their functional importance in extreme sulfur-rich environments in the deep sea.

Together, these examples from thermal springs, deserts, hypersaline lakes, and deep-sea vents illustrate how extreme environments expand our understanding of viral diversity, function, and adaptation (Table 2). Although these strikingly specialized but identifiable functions demonstrate that viruses can directly shape how their hosts respond to extreme environments, more than half (and in some cases up to 80%) of the viral genes reported in these studies remain without functional assignments. The biochemical functions of most of these viral proteins remain speculative, underscoring a pressing need for targeted experimental validation. Characterizing extremophile viral proteins could yield enzymes of substantial biotechnological interest, such as those exhibiting remarkable thermal stability, salt tolerance, or acid resistance. To harness this potential, interdisciplinary efforts combining metagenomics, structural biology, and biochemical experimentation are essential to illuminate the functional roles of viruses thriving at life’s extremes.

## 4 Challenges in the Characterization of Viral Proteins

### 4.1 Lack of Homologous Sequences

Viral proteins are incredibly diverse. Many viral genes are so divergent that standard sequence similarity searches (e.g., BLAST^96^ or MMseqs2 ^97^) find no meaningful hits in databases (Table 3 and Figure 3). Detecting distant evolutionary relationships from a sequence alone is difficult.^98,99^ Thus, a newly discovered viral protein often starts as an ORF with no known relatives, yielding no clues as to its function. For example, a novel phage gene might not match any domains or motifs with functional annotations in standard reference protein databases like Pfam,^36^ PHROG,^34^ VOGDB,^33^ eggNOG,^100^ or KEGG KOfam.^101^ This is compounded by the fact that viruses often evolve via rapid mutation, gene shuffling, or recruiting genes from hosts and then diverging them.^102,103^ Remote homology detection methods^104^ (profile HMMs, etc.) can sometimes classify these proteins into broad families, but even advanced clustering using remote homology as employed by the phage-specific PHROG database could only assign putative functions to ∼50% of viral protein families,^34^ leaving the rest labeled as “unknown function.”

**Figure 3:**
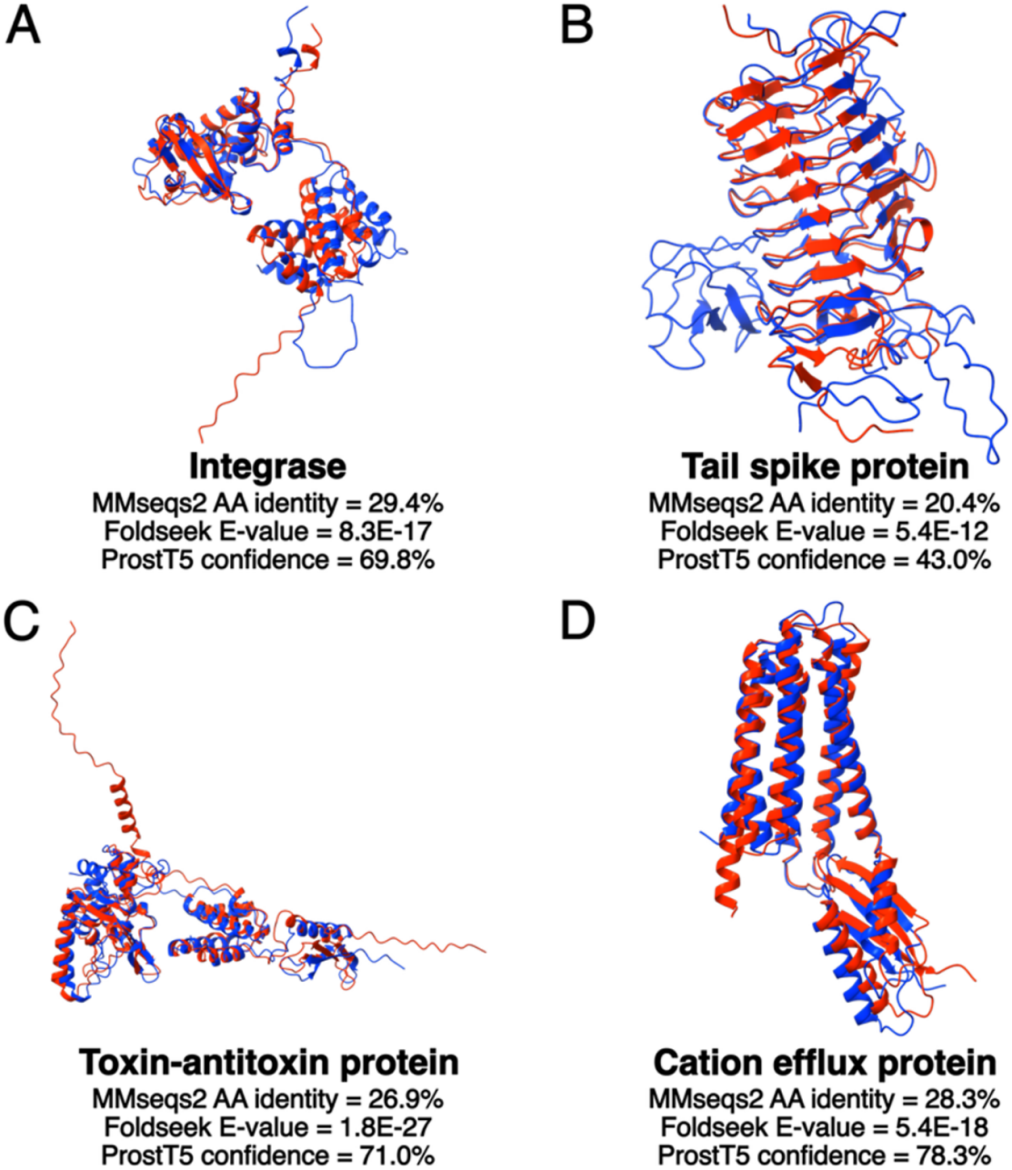
Protein structural similarity reveals functions missed by amino acid similarity. Protein structures predicted by ColabFold^105^ are shown for (A) a phage integrase (PHROG^34^ 1), (B) a tail spike protein (PHROG 3783), (C) a toxin–antitoxin protein (NetFlax^106^ WP_044886736.1), and (D) a cation efflux protein (PHROG 7308). Query proteins (red) were obtained from phage genomes identified in soil metagenomes. Structural alignment hits to reference phage proteins (blue), with Foldseek^99^ *E*-values and ProstT5 ^107^ confidence scores, were obtained using PHOLD.^108^ The amino acid (AA) identity between proteins was obtained with an MMseqs2 ^97^ search. These examples illustrate that sequence-based methods such as MMseqs2 often yield low-identity alignments with limited functional insight, whereas structure-based comparisons (e.g., PHOLD using Foldseek) reveal significant structural similarity to phage proteins with known functions.

**Table 3:** Key Challenges in Characterizing Viral Proteins and Potential Mitigation Strategies

challenge	summary	mitigation strategies
Lack of Homologous Sequences	Many viral proteins are so divergent that they have no detectable relatives in sequence databases, leaving no clues to their function	- Remote homology and clustering: Group unknown proteins into families to reveal distant relationships or conserved motifs (see refs 28,86 ).
		- Structure-based inference: Leverage structure-guided annotation tools that can infer function from structural features even when sequences differ (see, refs 108,111 Figure 3)
		- Protein language models: Use transformer-based models like ESM-2,^119^ ProstT5,^107^ or Protein Set Transformer (PST)^120^ to extract functional embeddings from sequences, enabling functional prediction without sequence homology (see refs 108,121 ).
Limited Structural Data	Very few novel viral proteins have solved 3D structures (most known structures are of common capsid or enzyme proteins), which hampers function prediction by fold comparison.	- AI-driven structure prediction: Use AlphaFold2 to model structures for uncharacterized viral proteins, expanding structural coverage (see, refs 110,112 Figure 3).
Misleading Homology-Based Annotations	Automated annotations can be incorrect – viral genes may be given enticing, but wrong functions based on weak similarity. Such misannotations propagate without manual curation or evidence.	- Stringent curation: Treat homology-based predictions with caution. Cross-check annotations against gene context and known biology; avoid assigning definitive functions without supporting evidence (see Supporting Information in ref 122).
		- Experimental validation: Prioritize lab experiments/tests for high-impact predictions. Validating a few cases helps correct errors and improve annotation accuracy (see examples inTable[Table tbl2]).
Dependency on Host Context	Several viral proteins may act only in the presence of their host or under specific conditions. If the host is uncultivated or the interaction is complex, it is difficult to deduce or test the protein’s function in isolation.	- Heterologous expression: Clone and express the viral gene in a model organism or cell-free system to test its activity *in vitro*, bypassing the need for the native host (see examples in Table[Table tbl2])
		- Genetic complementation: Introduce the viral gene into a host strain lacking the equivalent gene to see if it can restore the missing function, indicating a similar role. Conversely, delete the viral gene to observe effects on infection, linking the protein to a phenotype (see examples in Table[Table tbl2])
		- Environmental context assays: Leverage metatranscriptomics/metaproteomics to check if the viral gene is expressed under relevant conditions in natural samples (see refs 123,124 ). Use microcosm or mesocosm experiments (with labeled substrates, stable isotope probing) to see if the presence of the viral gene correlates with specific metabolic activities in a community setting (see refs 125,126 ).
Scale of Viral Dark Matter	The number of unknown viral genes far outpaces our capacity for one-by-one characterization. Traditional experiments are slow and usually limited to model viruses and hosts, making it infeasible to tackle the vast “viral dark matter” by conventional means alone.	- Prioritization of targets: Use computational analyses to narrow down which unknown viral proteins to study first (see refs 65,127 )
		- High-throughput screening: Develop scalable assays to probe many genes in parallel (see refs 118,128 ).

### 4.2 Limited Representation in Structural Databases

Structure can sometimes reveal function; for instance, a protein might have the fold of a protease or a kinase even if its sequence did not show it. But historically, very few viral hypothetical proteins have had solved 3D structures. Most solved virus protein structures are of well-known virion components (capsids and tail fibers) or enzymes (polymerases and lysozymes) from model phages or viral pathogens. Consequently, the extensive catalog of small, functionally enigmatic phage proteins, such as antihost factors and metabolic enzymes, remains severely underrepresented in structural databases like the Protein Data Bank (PDB).^109^ This structural gap significantly hampers functional inference via fold comparisons for viral dark matter proteins (Table 3). Recently, computational approaches like AlphaFold2 ^110^ have begun addressing this gap by predicting structures for thousands of viral proteins, as exemplified by the newly developed VFOLD database (within VOGDB)^33^ and the Big Fantastic Virus Database (BFVD),^111^ both of which specifically contain viral proteins overlooked by general databases.^106^ The BFVD, for instance, contains over 350,000 predicted viral protein structures, approximately 62% of which show no or very low structural similarity to existing structural databases like AlphaFold DB^112^ and the PDB.^109^ Moreover, until recently, searching a query against millions of predicted structures was computationally infeasible; tools like Foldseek now allow fast structure-based searches^99^ (Figure 3), but the accuracy of function inference from predicted structure still needs expert verification.

### 4.3 Misleading Homology-Based Functional Predictions

A growing concern in viral genomics is the misannotation of viral genes with attractive but incorrect functions (Table 3). High-throughput prediction pipelines can assign enticing labels to viral ORFs that turn out to be wrong upon closer scrutiny.^113^ Martin et al. warned that the rush to catalog AVGs (and AMGs in particular) has led to an “epidemic of misannotation”, where functions are predicted without sufficient manual curation or evidence.^7^ One prominent example is the misclassification of glycoside hydrolases (GHs) in viral genomes. Viral GH-like domains, while often annotated as polysaccharide-degrading metabolic enzymes potentially aiding host nutrition, frequently have structural or virulence roles unrelated to metabolism. For instance, phage tail fibers and baseplate proteins often incorporate GH domains to degrade host surface polysaccharides, facilitating viral entry.^114,115^ Similarly, phage endolysins, enzymes responsible for host cell wall degradation at the conclusion of the phage replication cycle, can be homologous to host metabolic enzymes such as chitinases or muramidases, but their role is strictly in host cell lysis rather than in nutrient breakdown.^114–116^ Structural studies have illustrated that chitinases, chitosanases, and phage lysozymes share conserved core folds despite minimal sequence similarity, highlighting the difficulty in accurately predicting their biological roles solely by homology.^114,115^ Overall, this is not to say that viruses do not truly encode *bona fide* GHs for organic matter decomposition, but homology-based functional predictions alone are insufficient to discern between metabolic and essential functions of viral proteins with GH domains.

### 4.4 Dependency on Host Context

Many viral proteins act by interacting with host proteins or metabolites. Consider how AVGs function: a phage protein might redirect a host regulatory pathway by binding to a host enzyme or altering its regulation. Determining such a function might require knowledge of the host target, which in turn might not be known or easy to test without the host system (Table 3). If the host is not *yet* cultivated (which is typically normal for environmental microbes), we cannot do traditional genetic experiments like knockouts or complementation to learn the protein’s role. This makes it challenging to design experiments, as one might not even know what substrate or condition to test. For instance, an AVG might only function at a specific infection stage,^117^ in concert with specific other viral/host factors,^77^ or in certain environmental conditions,^40^ which is hard to recapitulate in isolation. The substantial dependence on host context means that even if we can purify a viral protein, we might miss its true function if the assays do not mimic the right conditions.

### 4.5 Scale of the Problem

The vast number of sequenced and uncharacterized viral genes, now in the hundreds of millions, far exceeds our current ability to functionally characterize them (Table 3). While viral gene functions are ultimately constrained by what their hosts can support metabolically, this constraint alone provides insufficient resolution to assign specific functions to viral proteins. Even when host metabolic capabilities are well-characterized, we cannot reliably predict viral protein function solely on the basis of host context. AVGs, by definition, are not essential for viral replication and may serve diverse roles from metabolic supplementation to host manipulation and environmental adaptation that cannot be deduced from host genome content alone. Furthermore, viruses can encode functions entirely absent from their hosts, such as the toxin-antitoxin systems encoded by archaeal viruses mentioned in Section 3, above, and these actually help infected hosts outcompete uninfected strains, demonstrating that viral innovations can extend beyond host capabilities.

Historically, functional analyses have proceeded slowly, characterizing individual genes or phages one by one. Recently, high-throughput approaches such as deep mutational scanning, pooled selection assays, and CRISPR-based genome editing have begun addressing this bottleneck by systematically probing tens of thousands of phage variants simultaneously.^118^ However, these methods have primarily been limited to model phages and easily cultivable hosts such as *E. coli*.^118^ Extending such powerful approaches to diverse environmental phages and nonmodel hosts remains challenging due to technical hurdles in phage-host compatibility and library construction. Nonetheless, scaling these high-throughput functional genomics methods more broadly holds promise for systematically illuminating viral dark matter on a global scale.

### 4.6 Strategies to Characterize Auxiliary Viral Genes (AVGs)

Given the enormous scale of unknown viral proteins, prioritization is essential. Bioinformatic analyses can identify candidate genes of interest based on several criteria: (a) *abundance or ubiquity*—proteins that are highly abundant in metagenomes or occur in many samples, indicating ecological importance; (b) *genomic context*—for example, a viral gene adjacent to known metabolic genes may also have a metabolic function; (c) *phylogenetic or taxonomic scope*—proteins unique to viruses infecting certain hosts or environments, pointing to specialized functions; and (d) *Conservation*—unknown proteins that are conserved across many related viruses suggest an important role worth investigating.

Closing the gap between predicted function and confirmed activity for viral proteins will require a concerted increase in the level of experimental work. So far, logistical challenges have limited these efforts; many viruses with interesting genes infect hosts that are difficult or improbable to culture in the lab. Nevertheless, creative approaches can be employed to experimentally probe viral protein functions without needing the full virus-host system (see genes with validated functions in Table 2).

For example, if a phage genome harbors a candidate enzyme gene, researchers can synthesize the gene and express it in a model organism or cell-free system to test its activity *in vitro*. Advances in heterologous expression and protein engineering now make it feasible to produce many viral enzymes, even those from rare environmental phages (Table 2).^129^ Successful expression opens the door to biochemical assays: Does the protein catalyze the expected reaction? Is its activity measurable? One can be used to pursue structural biology (X-ray crystallography or cryo-EM) to glean mechanistic insights once the protein is purified. In the case of the soil viral chitosanase mentioned above, the investigators bypassed the need to culture the virus by cloning the gene, expressing the protein in *E. coli*, and determining its 3D crystal structure, which confirmed the anticipated active sites for chitosan hydrolysis (Table 2).^65^ This approach can be broadly applied to other viral enzymes of interest.

In addition to *in vitro* biochemistry, genetic experiments can illuminate function. Phage genes suspected to influence host metabolism could be tested by introducing them into a host strain lacking the corresponding native gene (a complementation test) to see if the viral gene can restore the function.^130^ For instance, if a phage carries a *folA* (dihydrofolate reductase, known to be involved in DNA precursor synthesis and folate metabolism) gene, one could knock out *folA* in the host and see if the phage-encoded version rescues growth. Similarly, if efforts to cultivate the host have been successful, one could knock out or inhibit the host’s gene during phage infection to see if the viral version compensates for the loss, demonstrating the phage protein’s functionality. Where possible, constructing mutant viruses that delete the gene and observing the effect on replication and host physiology are the most direct evidence of function, though this remains technically difficult for many environmental viruses.

Modern ‘omics and cell biology techniques also offer indirect routes to validation. Metatranscriptomics and metaproteomics can detect whether putative AMGs are actually expressed during infection in natural samples. For example, if a viral gene is highly expressed at the precise time it would be needed (say, a phage nitrogen metabolism gene expressed during host nitrogen starvation), that bolsters the case that it is functional in that context. However, a limitation with this approach is that these methods can detect expression only at the time of sampling; failure to detect expression may simply reflect the absence of the right environmental trigger and does not prove the gene is nonfunctional. Environmental context assays can help address this limitation (Table 3). Mesocosm and microcosm experiments, which maintain seminatural systems under controlled conditions, offer a useful intermediate between lab and field studies.

These partially controlled systems can be manipulated to introduce environmental or chemical changes that may elicit transcriptional or translational responses in microbial communities. When paired with genomics, transcriptomics, proteomics, or stable isotope probing, mesocosms and microcosms allow researchers to track viral gene expression and function following environmental shifts without needing to isolate viruses or hosts. Stable isotope probing (SIP) could be particularly valuable for testing AMG activity: by incubating a community with a labeled substrate (e.g., ^13^CO_2_) and tracing the incorporation of the label into biomolecules in the mesocosm/microcosm, researchers can assess whether a virus population carrying a specific AMG contributes to the associated metabolic process. For instance, if a virus encodes a methanogenesis-related gene, then SIP could confirm its functional role by showing label incorporation into methane or host biomass when the viral gene is present and expressed in the active community. Although challenging, similar ecosystem-level assays have recently been used to infer viral activity *in situ* (Table 2).^69,125,126^

To overcome the limitations of one-at-a-time gene characterization when faced with many promising candidates, emerging high-throughput strategies are beginning to make functional viromics more scalable and applicable to diverse environmental phages. For example, Chen et al. developed the PhageMaP method, which enables genome-wide interrogation of phage gene function by combining modular genome engineering with pooled phenotypic screens,^128^ allowing researchers to map essential, nonessential, and host-specific genes across multiple phages and bacterial hosts.^128^ This approach provides a flexible framework for functional analysis beyond the classic model systems. Similarly, Huss et al. developed the method Meta-SIFT to address the challenge of annotating proteins without known homologues by using deep mutational scanning data to identify conserved sequence motifs and functionally important residues in phage proteins.^131^ By integrating this with metagenomic data, Meta-SIFT can predict meaningful functional regions even in highly divergent proteins.^131^ Together, these methods offer promising paths to extend functional genomics to viral dark matter, enabling more systematic large-scale annotation of unknown viral proteins across ecosystems.

Overall, there is a rich toolbox available, from classical biochemistry to cutting-edge multiomics to probe viral protein function (Table 3). We deliberately refrain from prescribing an exact experimental workflow, as the optimal approach will differ case by case. The key point is that integrating experimental validation into viromics studies is essential. Even a few targeted validations can have an outsized impact: they provide “proof of concept” that certain viral genes are truly functional, help calibrate bioinformatic predictions, and sometimes reveal surprises that revise our understanding of viral capabilities. Going forward, collaborations between the bioinformaticians and microbial ecologists who identify candidate genes and the experimental biochemists who can test them will be especially powerful. By combining strengths, such cross-disciplinary teams can systematically chip away at the mountain of viral dark matter, one function at a time.

## 5 Outlook

### 5.1 Viral Dark Matter for Basic Science

Why should experimentalists invest their time in characterizing proteins encoded by uncultivated viruses? First, from the standpoint of fundamental science, viral dark matter holds deep insights into evolution and ecology. Viruses have had billions of years to sample sequence space and to evolve novel solutions to biological problems. Many protein families likely originated in viruses or have been extensively reshaped by viral evolution. By exploring viral proteins of unknown function, we may discover entirely new protein folds or biochemical activities that expand the known repertoire of life’s catalysts. Such discoveries can, in turn, illuminate how viruses manipulate hosts and influence ecosystem processes (see examples in Section 3). Filling in these gaps is key to accurately predicting ecological responses to change, such as in the context of human, animal, and plant diseases, climate-driven shifts in the oceans and lakes, or nutrient alterations in soil.

### 5.2 Viral Dark Matter for Biotechnology

Second, there is a strong practical incentive: viral proteins have already proven their value in biotechnology and medicine, and many more applications undoubtedly await. A historical case in point is the discovery of reverse transcriptase in retroviruses in 1970,^132,133^ which was a watershed moment in molecular biology. This viral enzyme, initially a puzzling novelty, became the cornerstone of recombinant DNA technology by enabling cDNA cloning and RT-PCR. Likewise, phages have yielded a treasure trove of enzymes that are now routinely used in the lab: DNA polymerases for PCR and DNA sequencing, DNA ligases for cloning, and RNA polymerases like T7 RNAP for *in vitro* transcription, all of which originate from phages.^42^ In medicine, phage enzymes have been developed as novel antimicrobials. Phage endolysins are enzymatic antimicrobials that can swiftly lyse specific bacteria, including drug-resistant strains, without harming beneficial microbes.^43,44^ Similarly, phage depolymerases (which are often glycosidases) show promise in breaking down bacterial biofilms in chronic infections.^134^ These successes underscore that viruses encode unique biochemistry that biotechnology can leverage. In short, today’s “hypothetical” viral proteins have the potential to become tomorrow’s biotech workhorses or drug leads.

### 5.3 Auxiliary Viral Genes for Bioengineering Phage Therapies

One especially promising avenue emerging from viral dark matter research is the exploitation of auxiliary viral gene AVGs in phage therapy. Because AVGs by definition are not required for the phage to reproduce under ideal laboratory conditions, they can often be added, removed, or modified without completely destroying the phage viability. This makes them attractive targets for phage bioengineering (Figure 4). One can potentially equip therapeutic phages with beneficial cargo genes or remove detrimental ones to create a more effective antibacterial agent. By creating a “cocktail” of phages, with each phage engineered to encode for a function that improves therapeutic outcomes (Figure 4A), researchers can selectively target multiple bacterial defenses or virulence traits simultaneously and thereby increase host killing while limiting the potential for resistance.

**Figure 4:**
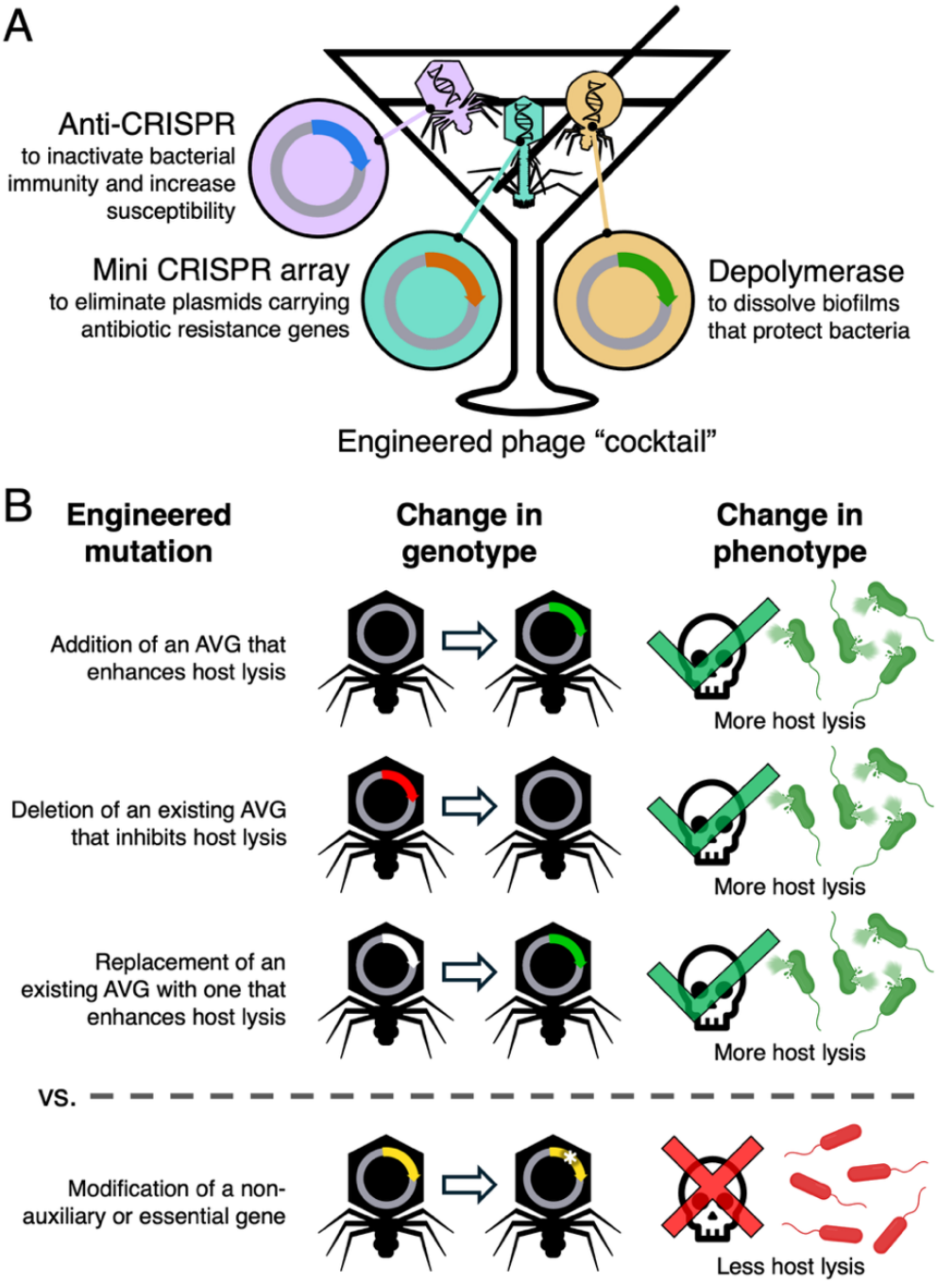
Auxiliary viral genes as targets for engineering phage therapies. (A) A hypothetical “cocktail” of engineered phages designed to improve phage therapy outcomes. Examples of auxiliary viral genes (AVGs) shown here include an anti-CRISPR protein to disable host immunity,^135^ a miniature CRISPR array that targets plasmids carrying antibiotic resistance genes,^136^ and a depolymerase that degrades biofilms and thereby exposes bacteria to more phages and antibiotics.^134^ (B) The benefits of targeting AVGs over nonauxiliary or essential phage genes. The top three rows illustrate that the addition,^135^ deletion,^128^ or replacement^137^ of AVGs can increase host lysis or susceptibility without disrupting core phage functions. In contrast, the bottom row shows that modifying nonauxiliary or essential genes can impair lysis or phage fitness^128^ and reduce therapeutic efficacy. Created in BioRender. Kosmopoulos, J. (2025) https://BioRender.com/8p12uxn.

One example is the use of phage-encoded anti-CRISPR (Acr) proteins. Anti-CRISPRs are small proteins produced by some phages (often carried in prophage elements) that inhibit the host’s CRISPR-Cas immune system, thereby protecting the phage from CRISPR-based defense.^138,139^ They were originally viral dark matter genes, tiny ORFs with no known function until they were discovered through clever screens. Dozens of Acr families have since been identified, each targeting different types of CRISPR systems.^127,140^ In the wild, not all phages have *acr* genes, but those that do have a clear advantage against CRISPR-proficient bacteria. Recognizing this, researchers have begun engineering phages to carry anti-CRISPR genes to make them more effective in therapeutic contexts. For instance, Qin et al. engineered a lytic phage of *P. aeruginosa* by inserting genes for AcrIF1–3 (which block the Type I–F CRISPR system of *P. aeruginosa*).^135^ The modified phage showed enhanced ability to replicate on bacteria with active CRISPR defenses and could thereby infect and kill strains that normally would fend off phages.^135^ In addition, these Acr-armed phages suppressed the emergence of phage-resistant bacterial mutants in experiments and even reduced antibiotic resistance in the bacterial population.^135^ This demonstrates a powerful principle: by leveraging an AVG (in this case, an anti-immunity gene), we can improve phage therapy outcomes against bacteria that were previously difficult to eradicate.

Crucially, using AVGs in engineering is often safer or more feasible than tinkering with essential phage genes (Figure 4B). If one tried to modify a phage’s capsid or replication proteins, the phage will likely lose viability. In contrast, adding a new AVG or swapping one AVG for another can often yield a viable phage because these genes are not strictly required for the phage life cycle in lab conditions. For example, phages can typically tolerate the insertion of a small gene like an anti-CRISPR or an antitoxin gene in their genome, as long as careful design ensures it does not disrupt other elements. Deleting an AVG that encodes an unwanted function (like a phage-encoded toxin that is undesirable in a therapy phage) can also be done without rendering the phage inert, since the gene is not needed for basic replication.

## 6 Call to Action: Harnessing Viral Dark Matter

Given these possibilities detailed above, we urge the scientific community, especially those with biochemical and molecular biology expertise, to engage with the challenge of viral dark matter. This is a call to broaden our perspective: viruses are not just vectors of disease or abstract sequences in databases but also reservoirs of unexplored functions. By partnering with virologists, microbial ecologists, and bioinformaticians, we can help turn putative annotations into proven activities. The payoff includes not only advancing our basic understanding of viral biology and ecology but also unearthing novel enzymes, therapeutics, and tools (Figure 5). Let us answer this call to action and illuminate the unknown viral functions that have waited too long in the shadows.

**Figure 5:**
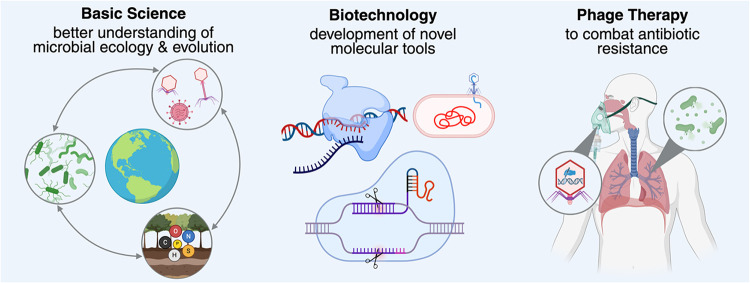
Applications for the study of viral dark matter. Uncovering and characterizing virus-encoded proteins, particularly those with metabolic or previously unknown functions, holds tremendous potential to advance basic science, enable the development of novel molecular tools, and support phage-based therapies to combat antibiotic-resistant infections. Created in BioRender. Kosmopoulos, J. (2025) https://BioRender.com/fsvku74.
